# Probing the magnetic profile of diluted magnetic semiconductors using polarized neutron reflectivity

**DOI:** 10.1038/s41598-017-06793-w

**Published:** 2017-07-24

**Authors:** X. Luo, L. T. Tseng, W. T. Lee, T. T. Tan, N. N. Bao, R. Liu, J. Ding, S. Li, V. Lauter, J. B. Yi

**Affiliations:** 1 School of Materials Science and Engineering, UNSW, Sydney, NSW 2052 Australia; 2 Bragg Institute, ANSTO, New Illawarra Road, Lucas Heights, NSW 2234 Australia; 3 Department of Materials Science and Engineering, National University of Singapore, 119260 Singapore, Singapore; 4 SIMS Facility, Office of the Deputy-Vice Chancellor (Research and Development), Western Sydney University, Locked Bag 1797, Penrith, New South Wales, 2751 Australia; 5 Quantum Condensed Matter Division, Neutron Sciences Directorate, Oak Ridge National Laboratory, Oak Ridge, Tennessee 37831 USA

## Abstract

Room temperature ferromagnetism has been observed in the Cu doped ZnO films deposited under an oxygen partial pressure of 10^−3^ and 10^−5^ torr on Pt (200 *nm*)/Ti (45 *nm*)/Si (001) substrates using pulsed laser deposition. Due to the deposition at relatively high temperature (873 *K*), Cu and Ti atoms diffuse to the surface and interface, which significantly affects the magnetic properties. Depth sensitive polarized neutron reflectometry method provides the details of the composition and magnetization profiles and shows that an accumulation of Cu on the surface leads to an increase in the magnetization near the surface. Our results reveal that the presence of the copper at Zn sites induces ferromagnetism at room temperature, confirming intrinsic ferromagnetism.

## Introduction

Weak ferromagnetism has been of importance in the last few decades for understanding the fundamental physical properties of new materials, such as defects induced ferromagnetism in nonmagnetic materials, ferromagnetism of antiferromagnetic nanoparticles and magnetic element doped semiconductors etc.^1–10^. Among these, diluted magnetic semiconductors (DMSs) are one of the typical examples of materials having weak magnetism^11, 12^. The original idea of DMS is to dope magnetic element into semiconductors to achieve both semiconductor and magnetic properties simultaneously^13^. Due to the very low concentration of magnetic element, the saturation magnetization of DMS is usually very low (less than 50 emu/cm^3^). Oxide based DMS, which is a very promising candidate for spintronic devices due to its high Curie temperature, has much lower magnetization than that of III-V based DMS^13^. Experimentally, magnetism is measured by a superconducting quantum interference device (SQUID), which has a very high sensitivity and resolution, whereas, it only provides the overall magnetization of the samples. No information on the origin of the ferromagnetism can be provided^14^. It is well known that currently, there is an intense debate on the origin of the ferromagnetism. Though room temperature ferromagnetism has been reported in various oxide semiconductor based DMSs doped with a variety of transition metals, such as, Mn^15^, Co^16, 17^, Fe^18^, and Ni^19^, Cu^20–23^ *etc*, some researchers argue that the ferromagnetism is not intrinsic but is contributed by extrinsic factors, such as secondary phase or doping clusters^24–26^, which are usually located in the interface or on the surface^27–29^. However, there is currently no strong evidence on this kind of ferromagnetism. Available technique for identifying the secondary phase or clusters is TEM/EDX by providing high resolution images or element mapping. In addition, it is not clear that the ferromagnetic phase is directly associated with the secondary phase or clusters. Furthermore, it has been argued^28^ that the clusters should demonstrate paramagnetic signal at room temperature due to their very small sizes. Moreover, the magnetic signal may also come from the contamination of substrate. Polarized neutron reflectometry (PNR) is one of the techniques, which can directly provide the depth profiles of the magnetic moment distribution in thin films or multilayers from the surface to the substrate^30^. However, due to the limitation of the sensitivity, there is no report on the magnetism of oxide based DMSs by PNR due to very weak magnetic signals in these materials. Cu doped ZnO has been one of the most interesting systems for oxide based DMSs since Cu has two valence states dependent on the preparation parameters, such as oxygen partial pressure for thin film fabrication^21^. Many research works have been performed to study the mechanism of the ferromagnetism. Experimental and theoretical works have shown that *Cu* ^2+^ has local moment and contributes to the ferromagnetism of Cu doped ZnO system^24, 31^. Though there is still debate on the origin of ferromagnetism, Herng *et al*. discovered an interesting phenomenon that the mutual manipulation between ferromagnetism and ferroelectricity can be achieved in 8% Cu doped ZnO film deposited on silicon substrate coated with Pt prepared by pulsed laser deposition^22^, which may introduce a new type material promising for the applications of multifunctional devices.

In this work, we used depth-sensitive PNR to probe the composition and magnetic depth profiles of the Cu-doped ZnO thin films deposited on Pt (200 *nm*)/Ti (45 *nm*)/Si (001) substrate at 873 *K* and obtained the details of the structure, element diffusion and magnetic distribution of thin films. Combining with other characterization, such as XRD (X-ray diffractometry), transmission electron microscopy (TEM), UV and X-ray photoelectron spectroscopy (XPS) and magnetic property measurement, we show that magnetization is strongly correlated to the Cu doping concentration.

## Experiment procedure

ZnO:Cu (8 *at* %) films were deposited on commercial Pt (200 *nm*)/Ti (45 *nm*)/Si (001) substrates (Kejing, China) by a pulsed laser deposition (PLD) system. The ZnO:Cu ceramic target was fabricated by mixing the powders of ZnO and CuO (99.99 *at*%, Sigma-Aldrich) with a proper ratio and subsequently sintered at 1123 *K* using a spark plasma sintering (SPS) technique. The sintered target was then annealed in air at 1073 *K* for removing the impurities of graphite on the surface. The deposition power of the laser is 180 *mJ* and the substrate temperature is at 873 *K*, which is for the better resistivity, in consistent with ref. 22. The thickness of the films was controlled to be 50 *nm*. The oxygen partial pressure (PO_2_) was varied from 10^−3^ to 10^−5^ torr in order to avoid the formation of dopants clusters or secondary phases^21, 22^. The crystal structure of the film was characterized by XRD with Cu K *α* radiation and TEM. The composition of samples was determined by XPS using a monochromatized Al K^*α*^ X-ray source (*hv*) of 1,486.6 *eV* with 20 *eV* pass energy. Electronic band gap was estimated by a UV spectrophotometer. Magnetic properties of the samples were investigated using a superconducting quantum interference device (SQUID; XL-7, Quantum Design, San Diego, CA, USA) and polarized neutron reflectometry (PNR) (SNS, Oak Ridge National Lab, USA). The element distribution along the depth of films was examined by secondary ion mass spectrometry (SIMS) (Cameca IMS 5FE7, France).

## Results and Discussion

**Figure 1:**
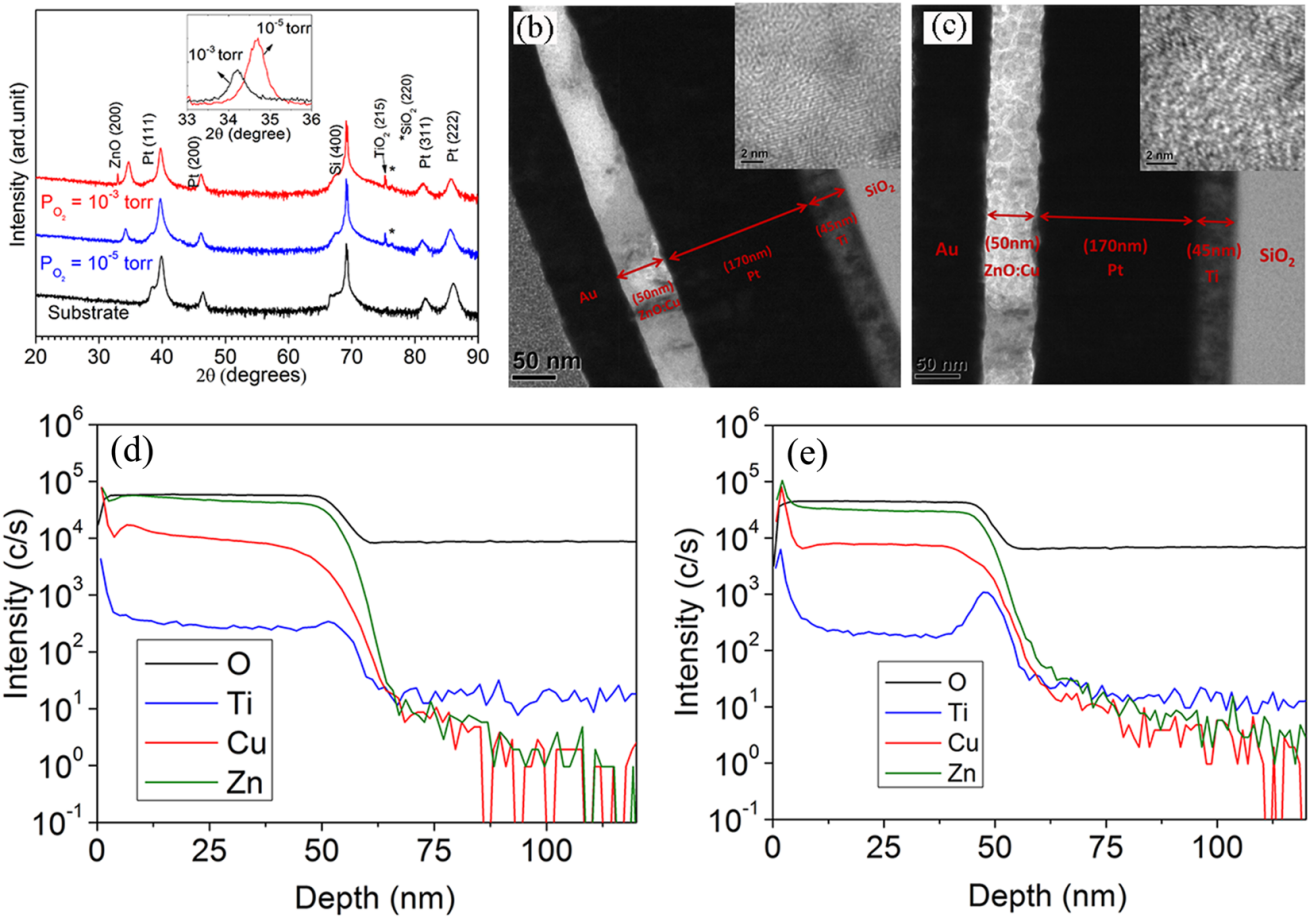
(**a**) XRD patterns of Cu-ZnO films deposited under PO_2_ = 10^−3^ torr and PO_2_ = 10^−5^ torr. The inset shows the enlarged region in a narrow scale close to (002) peak. TEM image of Cu-ZnO film deposited under (**b**) PO_2_ = 10^−3^ torr; (**c**) PO_2_ = 10^−5^ torr. The insets show the high-resolution TEM image of ZnO:Cu films. (**d**) and (**e**) are SIMS spectra of Cu-ZnO deposited at PO_2_ = 10^−3^ and 10^−5^ torr, respectively.

XRD spectra are used to examine the quality of crystal structure of Cu-ZnO samples and possibility of existence of secondary phases. From Fig. 1(a), there is only one peak corresponding to (200) of ZnO, indicating that both Cu-ZnO films deposited under an oxygen partial pressure of 10^−3^ and 10^−5^ torr respectively are highly textured with a wurtzite structure. However, besides the substrate signal, we also detected two kinds of second phases in the as-prepared samples, which are identified as SiO_2_ (440) and TiO_2_ (114). The appearance of SiO_2_ peak is due to recrystallization of amorphous SiO_2_ layer in the substrate during a high temperature deposition process. The formation of TiO_2_ should be caused by the oxidation of Ti atoms diffused from substrate during the sample preparation. Moreover, the crystal structure of TiO_2_ is identified as Brookite. From the inset of Fig. 1(a), it is noticed that peak position of ZnO in oxygen-deficient sample (PO_2_ = 10^−5^ torr) shifts to lower angle compared to oxygen-rich sample (PO_2_ = 10^−3^ torr). This indicates a larger lattice constant for the sample deposited at lower PO_2_. It is well known that ZnO films deposited under oxygen-deficient condition contains plentiful oxygen vacancies^15^. Those defects are favored sites for the clustering of CuO and TiO_2_. Those oxide clusters in nanoscale interstitially located in ZnO lattice lead to a larger lattice constant. More details will be discussed in the analysis of UV and SIMS spectra.

**Figure 2:**
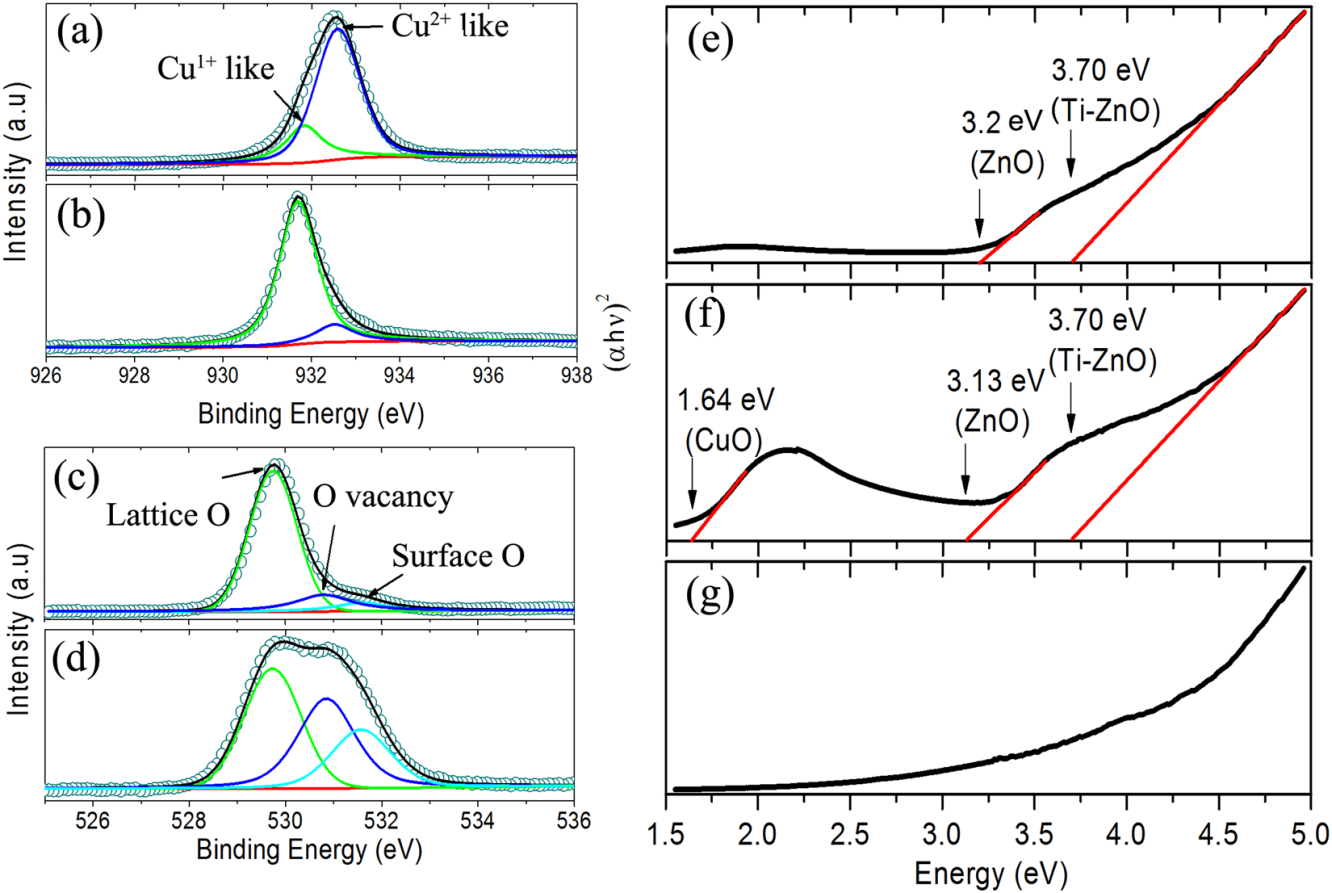
XPS spectra of ZnO:Cu films. (**a**) Cu 2p 3/2 edge, PO_2_ = 10^−3^ torr. (**b**) Cu 2p 3/2 edge, PO_2_ = 10^−5^ torr. (**c**) O 1 s edge, PO_2_ = 10^−3^ torr. (**d**) O 1 s edge, PO_2_ = 10^−5^ torr. (**e**) (*αhV*)2 versus photon energy plots of ZnO:Cu films at PO_2_ = 10^−3^ and 10^−5^ torr, respectively.

Figure 1(b) and (c) show the TEM images of the Cu-ZnO films deposited under an oxygen partial pressure of 10^−3^ and 10^−5^ torr. It can be seen that both films have thicknesses of approximately 50 *nm*. The thickness of Pt is around 170 *nm*. Below Pt, there is a layer of Ti film with a thickness of approximately 45 *nm*. The insets are the high resolution TEM images of the corresponding films. Both low and high resolution TEM images show the polycrystalline structure of the films. However, from XRD spectra, only (002) peak of ZnO can be observed, suggesting the highly textured growth of the thin films. Figure 1(d) and (e) depict SIMS data of the samples deposited under PO_2_ = 10^−3^ and 10^−5^ torr. The depth profile is consistent with the layered structure of the cross-section shown in the TEM image (Fig. 1(b) and (c)). According to the SIMS result, Ti atoms have diffused into Cu-ZnO film during the deposition process. In addition, it is observed that the distribution of Cu is non-uniform and a large amount of Cu atoms diffuse onto the surface and the doping concentration of Cu decreases gradually from the top of the film to the interface between ZnO and Pt substrate. To understand the composition and chemical state of samples, the XPS spectra of Zn 2p3, Cu 2p and O 1 s core levels were measured. The binding energy of the peaks was calibrated by taking the C 1 s peak (284.7 *eV*) as a reference. Figure 2(a) indicates that the change of oxygen environment during deposition has a negligible impact on the chemical state of Zn. In contrast, the chemical state of Cu is very sensitive to the oxygen partial pressure. From a close inspection of XPS peaks of Cu 2p3/2 (Fig. 2(b), the majority of Cu ions in oxygen-rich sample are in d9 (Cu^2+^-like) state, while d _10_ (Cu^1+^-like) state is dominant in the oxygen-deficient sample. A similar result is also reported by Herng *et al*.^21, 22^. Nevertheless, The difference of the two samples of XPS spectra is also reflected on the O 1 s edge as shown in 3(c). The blue dissociated peak, located at 530.8 *eV*, corresponds to oxygen deficiency in ZnO system, such as oxygen vacancies^15^. The integrated area under the blue peak increases significantly at PO_2_ = 10^−5^ torr, indicating a higher concentration of oxygen vacancies in oxide-deficient sample. The cyan peaks centred at 531.7 *eV* are assigned as the absorbed or dissociated oxygen on the surface^32, 33^. The increase of the peak intensity may suggest increasing amount of defects on the surface of the oxygen-deficient sample.

**Figure 3:**
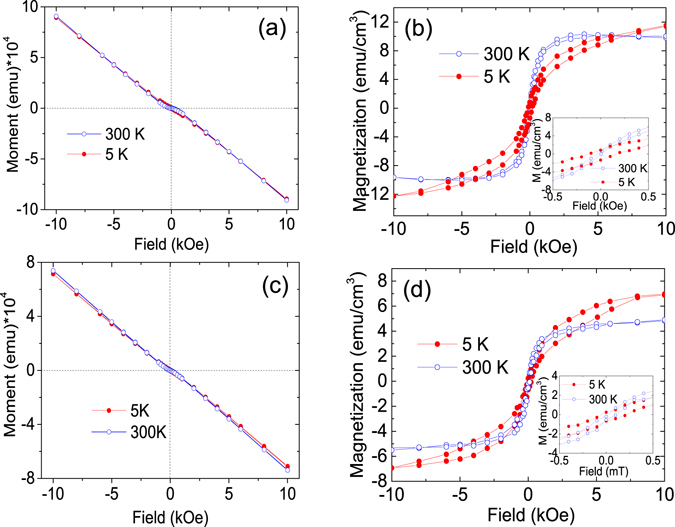
M-H loops for Cu-ZnO films. (**a**) Original curves without the subtraction of substrate signal for PO_2_ = 10^−3^ torr sample. (**b**) M-H curve after the substraction of substrate signal of (**a**). (**c**) Original curves without the subtraction of substrate signal for PO_2_ = 10^−5^ torr sample. (**d**) M-H curve after the subtraction of substrate signal of (**c**).

To investigate the influence of dopant and oxygen vacancies on the band structure of ZnO system and verify the existence of second segregation, UV measurements were performed for both oxygen-rich and oxygen-deficient samples and the substrate is as a reference. The results are shown in Fig. 2(e)–(g). For the sample prepared under high-oxygen environment (10^−3^ torr), the band gap of ZnO is 3.20 *eV*, which is slight smaller than the standard value (3.4 *eV*) due to the strong O(p)-Cu(d) exchange interaction^34^. From this perspective, the narrower band gap also indicates that the effective Cu doping has been achieved in our experiments. There is another phase that can be observed in the samples with the band gap of 3.71 *eV*. It should belong to the phase of Ti doped ZnO (TZO)^35^. The formation of TZO is mainly due to the diffusion of Ti atom from the substrate during the high temperature deposition process. SIMS analysis has confirmed the diffusion of Ti in ZnO film. For the oxygen-deficient sample (10^−5^ torr), the band gap of Cu-ZnO is approximately 3.13 *eV*, which is lower than that of the oxygen-rich sample. It may be due to a large number of electron carriers induced by oxygen vacancies. Interestingly, apart from TZO phase, additional second phase of CuO is found in oxygen-deficient sample. The band gap is measured as 1.64 *eV* ^36^. The reasonable interpretation is that the high concentration of oxygen-deficient defects makes the doped crystal difficult to form a stable solid solution in such high-doping concentration (8%) samples. These oxygen-deficient regions created during deposition then become the favourable sites for the formation of Cu oxide cluster. From XRD analysis, CuO phase has not been detected. It may be either due to its small amount beyond the detection limit of XRD analysis or it exists in nanoscale clusters with strong disordering. In addition, from previous XPS results, d9 (Cu^1+^-like) is dominant in oxygen-deficient sample rather than d10 (Cu^2+^-like), suggesting that CuO phase is negligible and the majority of Cu is successfully doped in ZnO lattice. It should be noted that the bandgap of SiO_2_ cannot be observed due to its large value (6.3 *eV*). The magnetic properties of the samples, were probed by SQUID both at 300 and 5 *K*. Figure 3(a) and (c) show the original data of the samples deposited under oxygen pressure PO_2_ = 10^−3^ and PO_2_ = 10^−5^ torr before subtracting the substrate signal, whereas Fig. 3(b) and (d) display the M-H loops of Cu-ZnO films after subtracting the substrate signal. It can be seen that both samples exhibit ferromagnetic ordering at room temperature as clear hysteresis loops with small coercivities are observed. Different from previous work of Cu-ZnO deposited under SiO_2_ substrate, a lower oxygen pressure leads to a decrease in the saturation magnetization. As seen from Fig. 3(b) and (d), the sample has a saturation magnetization of 9 emu/cm^3^ for films grown at PO_2_ = 10^−3^ torr, while the saturation magnetization becomes 4.7 emu/cm^3^ when the PO_2_ = 10^−5^ torr. At 5 *K*, both samples show a slight increase in the saturation magnetization. A small diamagnetic signal observed in the sample deposited under PO_2_ = 10^−3^ torr may be related to the secondary phase such as TZO, as described in the UV and XRD analysis or the antiferromagnetic coupling of Cu dopant for the high doping concentrations of Cu. A small increase of the saturation magnetization at 5 *K* compared to 300 *K* confirms the ferromagnetic ordering in the samples.

To verify the influence of the substrate on the magnetic properties, Cu-ZnO films with identical growth condition were deposited on quartz substrates. The SQUID measurements show that the films deposited on quartz under both high and low PO_2_ have a much lower saturation magnetization, 1.4 and 2 emu/cm^3^, respectively, compare to those deposited on Pt bottom layer. It suggests that substrate plays an important role in the ferromagnetic properties.

**Figure 4:**
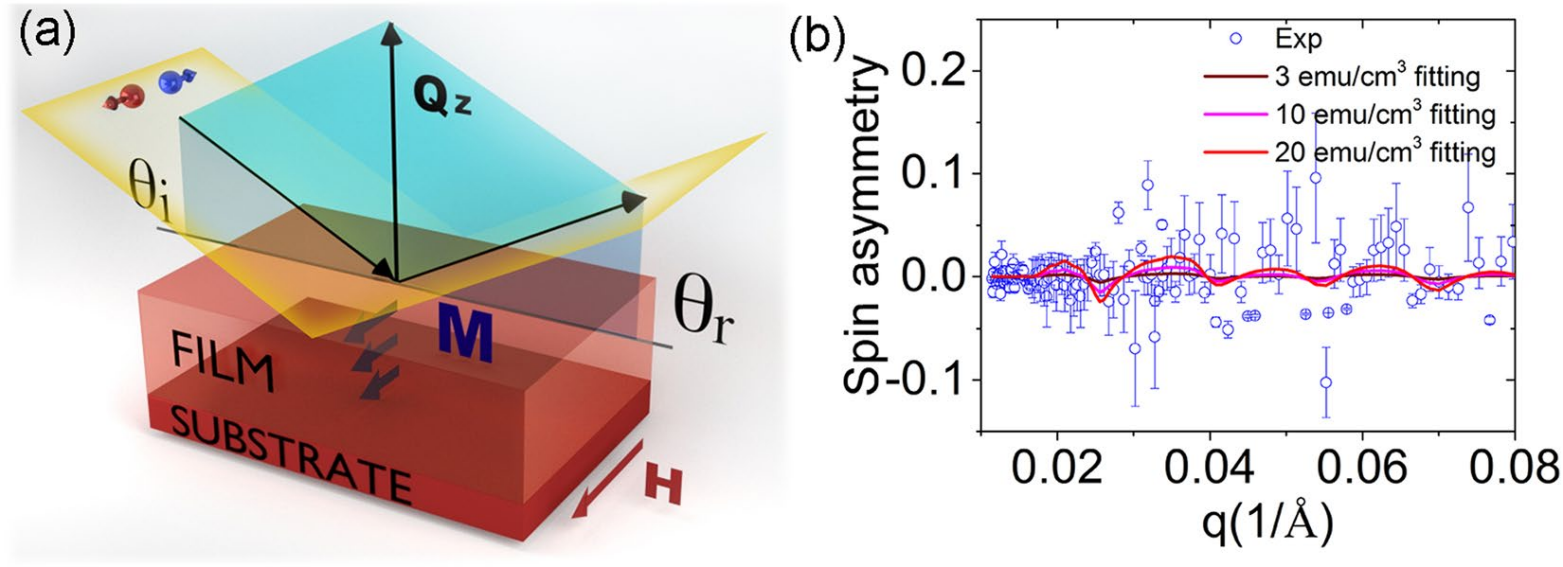
(**a**) A Scheme of PNR measurement. (**b**) The spin asymmetry of PO_2_ = 10^−3^ sample fitted with three different magnetization values.

**Figure 5:**
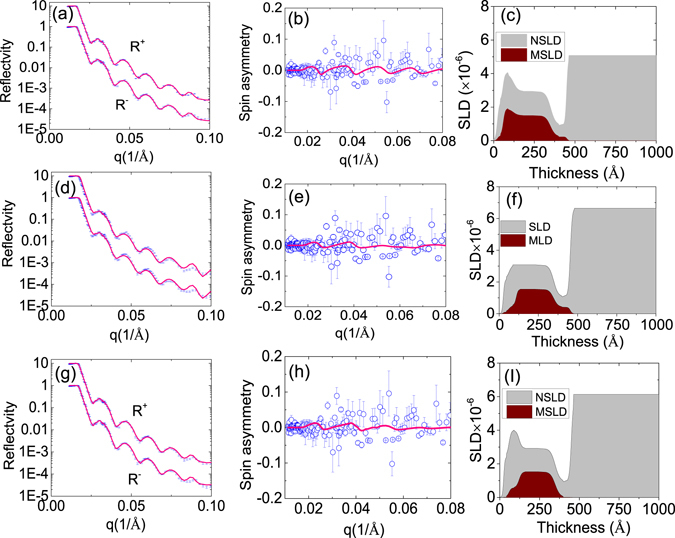
PNR data of Cu-ZnO deposited under an oxygen partial pressure of 10^−3^ torr with different types of fittings. The results of the best fit are presented in the top panel for R+ and R- reflectiviity, (**a**). Spin asymmetry (**b**). NSLD/MSLD profiles with nonuniform fittings (**c**). The experimental data are shown with blue circles with error bars and the fitting curves are shown with the red solid lines. In order to see clearly, the Y axis of MSLD has been multiplied by 50 times. The middle and the bottom panels confirm that the fit with a uniform NSLD/MSLD (**d**),(**e**),(**f**) and the nonuniform fittings of NSLD and unform MSLD (**g**),(**h**),(**i**) doe not provide a good agreement with the experimental data (solid lines deviate from the data from 0.07 < q < 0.1 in (**d**) and (**g**)).

To explore the origin of the ferromagnetic order and to directly probe the magnetization depth profile, we performed magnetic measurement by polarized neutron reflectometry (PNR)^37, 38^. PNR is a depth sensitive technique and allows to examine simultaneously the depth profiles of the distribution of the absolute value of magnetic moment and of the chemical composition from the film surface down to the interface with the substrate^39–41^, thus enables to fathom out whether the diffusion of Ti atoms or the distributions of dopants are attributed to the ferromagnetism. PNR experiments were performed on the Magnetism Reflectometer at Oak Ridge National Laboratory^42^. The high polarization (98.5%) of the neutron beam and a low background at the instrument are essential for reaching the sensitivity necessary to detect small magnetic moments^43, 44^. The reflected intensity is measured as a function of the momentum transfer, q = 4 *π* sin *θ*/*λ*, for two neutron polarizations R+ and R−, with the neutron spin parallel (+) or antiparallel (−) to the direction of the external field, H_*ext*_. In order to separate nuclear and magnetic components and to amplify the magnetic scattering, the spin-asymmetry SA = (R^+^ − R^−^)/(R^+^ + R^−^) is used. A value of SA = 0 corresponds to a zero net magnetic moment in the system. The schematic drawing of the PNR experiment and the results for the sample deposited at PO_2_ = 10^−3^ torr are shown in Figs 4(a) and 5, respectively. The fit to the data is performed simultaneously for R^+^ and R^−^ reflectivities using supermatrix formalism^45^. The results are shown in the top row in Fig. 5(a),(b) and (c). From the fit to the data, the distribution of the chemical depth profile nuclear scattering length density (NSLD) and magnetisation (Magnetic scattering length density MSLD) is obtained (presented in Fig. 5(c) with grey color and wine colors, respectively). It shows that the chemical and magnetic distributions are not uniform. At the interface between the Cu-ZnO and the Pt films there is a kink of a low NSLD. It is known that the elements in the sample have positive bound coherent scattering lengths, such as Zn (5.680 *fm*), O (5.803 *fm*), Pt (9.60 *fm*) and Cu (7.718 *fm*) as indicated in the brackets, except Ti, whose bound coherent scattering length is −3.438 *fm* (1 *fm* = 1 × 10^−15^ m). SIMS analysis confirm that Ti is accumulated in the interface area, thus directly corroborating our PNR results that the Cu-ZnO/Pt interface is enriched with Ti. Another noticeable feature is that a layer with a high NSLD was detected at the surface of Cu-ZnO film. In order to verify this finding and to test the sensitivity of the fit to this layer, we fitted the experimental results with two more models, as shown in Fig. 5. (I) Nonuniform NSLD and relatively uniform MSLD (Fig. 5d,e and f). (II) Uniform NSLD and MSLD (Fig. 5(g),(h) and (I)). From the corresponding results, we can see that the fitting of reflectivity curves and the spin-asymmetry fit are not good (see Fig. 5 middle and bottom rows), where the fitting curves considerably deviate from the experimental data points for q between 0.07 and 0.1 *Å* ^−1^. Hence, the best fit to the data reveals that the surface layer with high NSLD has also high MSLD. The high NSLD on the surface may be due to a high concentration of Cu. Using a conversion coefficient 1 emu/cm^3^ = 2.9 × 10^−9^ *Å* ^−2^ from the MSLD profile, we can obtain the magnetization of the film about 10 emu/cm^3^, which is consistent with SQUID measurement. As the magnetization profile has the same trend as that of Cu distribution (Fig. 1(e)), thus suggesting that Cu concentration determines the magnetization. To validate the result, we also show the spin asymmetry simulated with three assumed magnetization values (Fig. 4(b)). A bigger magnetic moment of 20 emu/cm^3^ evidently deviates from the experimental data points. For the simulation with magnetization value of 3 emu/cm^3^, the simulated spin asymmetry curve is almost flat. Hence, the magnetization determined from the PNR should be between 3 and 10 emu/cm^3^.

**Figure 6:**
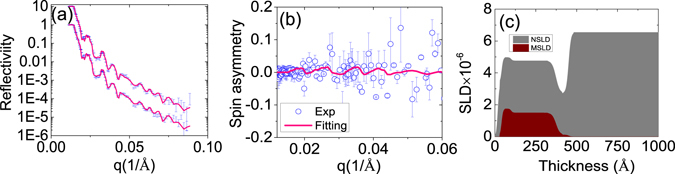
(**a**), (**b**), and (**c**) are spin reflectivity, spin asymmetry and NSLD and MSLD profile of the sample deposited under PO_2_ = 10^−5^ torr. The solid line (magenta) is the fitting curve and the circles represents experimental data (blue). In order to see clearly, the Y axis of MSLD has been multiplied by 50 times.

Using the same strategy, we obtained the NSLD and MSLD profiles of the sample deposited at PO_2_ = 10^−5^ torr as shown in Fig. 6. The best-fitted saturation magnetization is approximately 10 emu/cm^3^, slightly higher than the value of 5 emu/cm^3^ obtained from the SQUID measurement. The structure and magnetic profiles are slightly different from PO_2_ = 10^−3^ torr sample. In the middle of the film, the magnetization curve is flat, consistent with the distribution of Cu (Fig. 1(a)), confirming the role of Cu in the structure and magnetic profile. It should be noted that Ti also has a high concentration on the surface. However, it should not contribute to the enhanced magnetization on the surface since for PO_2_ = 10^−5^ sample, Ti also is high in the interface, while PNR analysis did not show enhanced magnetization in the interface of this sample, supporting that Cu concentration contributes to the enhanced magnetization.

## Conclusion

We have grown Cu-ZnO thin films (50 *nm*) on the electrode substrate using a PLD system under an oxygen partial pressure of 10^−3^ and 10^−5^ torr. Both samples show room temperature ferromagnetism. Due to a relatively high substrate temperature, Cu and Ti diffusion has been observed and the diffusion has strongly affected the structure and magnetic properties of the films. Combined with SIMS analysis, PNR results indicate that the distribution of magnetic moment is not uniform. The surface has a higher magnetic moment than other areas, due to the high Cu concentration by diffusion. Hence, we observed that Cu substitution leads to room temperature ferromagnetism, confirming the intrinsic ferromagnetism of Cu doped ZnO system.
